# Untargeted metabolomics reveals the effect of rearing systems on bone quality parameters in chickens

**DOI:** 10.3389/fgene.2022.1071562

**Published:** 2023-01-04

**Authors:** Dongfeng Li, Yongfu Wu, Kai Shi, Minghui Shao, Ying Duan, Minli Yu, Chungang Feng

**Affiliations:** College of Animal Science and Technology, Nanjing Agricultural University, Nanjing, Jiangsu, China

**Keywords:** chickens, bone quality parameters, rearing systems, metabolomics, LC-MS/MS

## Abstract

The objective of this study was to investigate the effects of rearing systems on the bone quality parameters in chickens using a metabolomics strategy. A total of 419 male one-day-old chicks were randomly allocated to two groups, a floor rearing group (FRG, *n* = 173) and a cage rearing group (CRG, *n* = 246). At 6, 8, 10, and 12 weeks of age, all chickens were radiographed by a digital X-ray machine, and body weight was recorded. At 12 weeks of age, 12 birds were selected from each group to obtain tibia and femur, and bone quality parameters of bone mineral density (BMD), mineral content (BMC), breaking strength (BBS), stiffness, Young’s modulus (YM), ash content, calcium content, and phosphorus content were determined. An untargeted metabolomics assay was performed to identify changes in the serum metabolic profile (*n* = 8 birds/group). The results showed that cage-reared chickens had wider tibiae and greater body weight compared with floor-reared chickens. There were no significant differences in BMC or BBS between the two groups (*p* > 0.05), but BMD, ash content, calcium content, and phosphorus content of the tibia and femur of FRG were significantly higher than those of CRG (*p* < 0.05). Greater stiffness and YM of the femur were also observed in birds raised in the FRG compared with those raised in the CRG (*p* < 0.05). Taken together, the results suggest that rearing systems affected bone quality parameters. Furthermore, 148 and 149 differential metabolites were identified in positive and negative ion modes by LC-MS/MS analysis, among which 257 metabolites were significantly correlated with 16 bone quality parameters, including leucine, myristoleic acid, glycocholic acid, and N-phenylacetamide. KEGG analysis indicated that 15 metabolic pathways, including six pathways of amino acid metabolism, two pathways of lipid metabolism, and two pathways of carbohydrate metabolism, were responsible for bone quality. Overall, the present study demonstrated the effect of rearing systems on bone quality parameters, and identified several metabolites and metabolic pathways associated with bone quality parameters.

## 1 Introduction

In recent years, the conventional cage rearing system for chickens has rapidly developed due to its high production efficiency and stocking density, helping the poultry industry to see an increase in overall revenue. Birds housed in cage rearing system had higher body weight and weight gain, the best values of feed conversion and European broiler index were also shown in cage-reared birds (Abo Ghanima et al., 2020). Cage-reared birds have restricted space and opportunities for walking, running, jumping, or wing-flapping. Meanwhile, high-intensity genetic selection for fast growth has an adverse effect on the respiratory, reproductive, and skeletal systems of the birds (Rawlinson et al., 2009). Therefore, birds living in cages face increased incidence of bone weakness, particularly leg problems (Talaty et al., 2010). Surveys of the walking ability of broilers found prevalence of 77.4% and 38.1% of impaired walking ability (gait score >0), and the prevalence of severe lameness (gait score >2) was 5.5% and 2.5% in the conventional and organic production systems, respectively (Tahamtani et al., 2018).

Bone density, which is the weight of minerals per unit volume of bone (g/cm^2^), is determined by the amount of mineral atom deposition within the bone matrix and the porosity of the bone matrix. Bone ash can be used to assess the degree of mineralization. Chickens with bone disorders usually have a lower percentage of bone ash (Shim et al., 2012). Young’s modulus (also referred to as the tensile modulus) is a measure of the mechanical properties of linear elastic solids. The modulus provides information about the tensile elasticity of a material (ability to deform along an axis).

Bone quality parameters such as bone breaking strength (BBS), bone mineral density (BMD), bone mineral content (BMC), and bone ash content have been utilized to assess the bone status of poultry (Kim et al., 2006; Rubin et al., 2007; Li et al., 2020; Ma et al., 2020), which are affected by the type of housing system (Regmi et al., 2015), genetics (Guo et al., 2017), nutrition (Ye et al., 2021), diseases (Liu et al., 2021), and age (Sanchez-Rodriguez et al., 2019). A recent study indicated that the breaking strength, percentage of the cortical area, and cortical thickness of the tibia, femur, and humerus of ducks raised in a floor system were significantly higher than in cage-reared ducks (Yang et al., 2022). Methods such as dietary changes in the calcium-phosphorus ratio, rearing in a range-free system with more opportunities for walking and flighting, increasing load-bearing exercise, and genetic selection for bone quality have been shown to have positive effects on poultry bone quality parameters (Dunn et al., 2007; Hester et al., 2013; Casey-Trott et al., 2017; Zhu et al., 2018).

Metabolomics is a new discipline featuring simultaneous qualitative and quantitative analysis of all small molecule metabolites of a certain organism or cell during a specific physiological period (Goodacre et al., 2004). Metabolomics also provides insight into metabolism in healthy animals to the point that pathogenesis is discovered during diseases (Lisuzzo et al., 2022). Shi et al. (2022) utilized untargeted metabolomics to illuminate the effect of genetic selection on skeletal muscles from two chicken lines and found that glycerophospholipid metabolism functioned in muscle flavor and development. Researchers have found a number of dietary icariin-induced differential metabolites highly correlated with both femur and tibia BMD in cage-reared hens (Huang et al., 2020).

In the present study, the developmental differences of the tibia at different periods were identified between meat-type chickens raised under cage and floor rearing systems, and bone quality parameters of the tibia and femur were measured. At the same time, we also analyzed the serum metabolomic profiles of chickens to identify metabolites and metabolic pathways associated with bone quality.

## 2 Materials and methods

### 2.1 Birds, management and sampling

All the study subjects and experimental procedures were reviewed and approved by the Animal Ethical and Welfare Committee of Nanjing Agricultural University. The population in this experiment was provided by Jiangsu Lihua Animal Husbandry Co., Ltd. (Changzhou, China). Birds were randomly collected from the offspring of eight males and 72 females of Chinese indigenous yellow-feathered meat-type specialized strain, which have been selected for body weight and feed conversion ratio for over five generations in the experimental station and the breeding goal is to be used as a paternal line. Briefly, before moving the eggs into the incubator, the weight of each egg was strictly controlled between 55 and 61 g, and the average value of incubated eggs was 57.70 g, the standard deviation was 1.72 g, and the coefficient of variation was 2.97%. All male one-day-old chickens were randomly allocated into two enclosed environmental poultry houses, a cage rearing group (CRG, *n* = 246) and a floor rearing group (FRG, *n* = 173). The progenies of eight males and 75 females were present in both groups. The birds raised on the floor had a space allocation of 1,000 cm^2^/bird. The cage rearing system adopted three-tiered stepped cages with dimensions 125 × 100 × 50 cm (approximately fifteen birds per cage). All birds were fed *ad libitum* with the same diets (Dietary Nutrients are shown in Supplementary Table S1). At 6, 8, 10, and 12 weeks of age, the body weight (BW) was recorded. Then all chickens were hung on a lateral plane equipped with an aluminum calibration step-wedge for radiography by a digital X-ray machine (HX-100P, Jiaxinhuixiong Technology Co., Ltd., Beijing, China), and the parameters were set as follows: voltage at 60 Kv, current per second at 3.2 mAs. Twelve birds of similar weight were selected from each group for bone measurement at 12 weeks of age. Before exsanguination, eight birds from each group were selected from the above groups; blood samples were collected from the wing vein and centrifuged at 3,000 r/min for 10 min at 4°C to obtain serum. After exsanguination, the right tibia and femur were carefully removed from the muscles and connective tissue with a scalpel and were stored at −20°C until analysis.

### 2.2 Tibia morphology parameters assessment

ImageJ software (version 1.8.0.112) was utilized to assess tibia morphology parameters, the specific usage was as follows: a straight line was drawn from the proximal to distal extremities of the tibiotarsus to determine tibia length, and another line was drawn at the middle vertical position of the first straight line to indicate the tibia midpoint diameter.

### 2.3 Mechanical testing of the tibia and femur

After the bones were thawed, BMD and BMC were determined with dual-energy X-ray absorptiometry *via* an InAlyzer (MEDIKORS, Korea) using a procedure according to the manufacturer’s introductions. Measurements of biomechanical properties were performed on a universal material testing machine (LR10K Plus, Lloyd Instruments Ltd., United Kingdom) by the three-point bending test. The distances between the two fulcrum points were 9 cm for the tibia and 7 cm for the femur. The results were read using NEXYGENPlus Data Analysis Software.

### 2.4 Determination of ash, Ca, and P content in bones

After measuring bone strength, all fragments of broken bones were collected to determine the percentage of ash based on the fat-free dry weight. The bones were dehydrated by drying at 105°C for 24 h and then defatted with petroleum ether for 48 h. After extracting the water and fat, the samples were dried at 105°C to constant weight in an oven (GPL-70, Labotery instrument equipment Co., Ltd., Tianjin, China). Ash content was determined after 24 h incineration at 600°C in an electric furnace (DK-98-II, Taisite instrument Ltd., Tianjin, China). Then, the bone ash content was expressed as the ratio of ash weight to fat-free dry weight. EDTA complexometric titration and ammonium molybdate spectrophotometric methods were adopted to determine the contents of calcium and phosphorus in the ash (Moss, 1961; Chanda et al., 2014).

### 2.5 Preparation of serum samples for metabolomics

All serum samples were thawed slowly at 4°C, and 100 μl aliquots were resuspended with 400 μl prechilled 80% methanol by vortexing. After being incubated for 5 min on ice, all samples were centrifuged at 14,000 × g for 20 min at 4°C. Samples of the supernatants were diluted to a final concentration containing 53% methanol by LC-MS grade water. The samples were transferred to fresh Eppendorf tubes and centrifuged at 14,000 × g for 20 min at 4°C for LC-MS/MS analysis. To ensure the reliable validation for metabolomic detection, quality control (QC) samples were conducted generally. Three pooled QC samples were obtained by mixing equal aliquots of all the samples.

### 2.6 LC-MS/MS analysis

LC-MS/MS analysis was performed using a UHPLC (1,290 Infinity LC, Agilent Technologies) coupled to a quadrupole time-of-flight (AB Sciex TripleTOF 6,600) from Shanghai Applied Protein Technology Co., Ltd. (Shanghai, China). The samples were placed into a 2.1 mm × 100 mm ACQUIY UPLC BEH 1.7 μm column (Waters, Ireland) for HILIC separation. The mobile phase contained 25 mM ammonium acetate and 25 mM ammonium hydroxide in water (A) or acetonitrile (B) in both ESI positive and negative modes. The gradient was 85% B for 1 min and was reduced linearly to 65% during 11 min, and then was reduced to 40% for 0.1 min and kept constant for 4 min, then increased to 85% for 0.1 min, with a re-equilibration for 5 min. An ACQUIY UPLC HSS T3 1.8 μm column (Waters, Ireland) was used for RPLC separation. The mobile phase contained water with 0.1% formic acid (A) and acetonitrile with 0.1% formic acid (B), and 0.5 mM ammonium fluoride in water (A) and acetonitrile (B) in ESI positive and negative mode, respectively. The gradient was 1% B for 1.5 min and was linearly increased to 99% during 11.5 min, then kept constant for 3.5 min, and reduced to 1% for 0.1 min, with a 3.4-min re-equilibration period. The gradients were kept at a flow rate of 0.3 ml/min, and the column temperatures were kept constant at 25°C. A 2 μl aliquot of each sample was injected for each run.

The ESI source conditions were set as follows: Ion Source Gas 1 at 60 psi; Ion Source Gas 2 at 60 psi; curtain gas (CUR) at 30 psi; source temperature, 600°C; IonSpray Voltage Floating (ISVF), ±5500 V. In the MS period, the instrument was set to acquire over the m/z range of 60–1,000 Da, and the accumulation time for TOF MS scan was set to 0.20 s/spectrum. In the MS/MS period, the instrument was set to acquire over the m/z range of 25–1,000 Da, and the accumulation time for the product ion scan was set to 0.05 s/spectra. The parameters of the product ion scan were set as follows: collision energy (CE) at 35 V with ±15 eV, decluttering potential (DP) of 60 V (+) and 60 V (−) in positive and negative ion modes, excluding isotopes within 4 Da, and 10 candidate ions to monitor per cycle.

### 2.7 Metabolomic data analysis

Filter out undetected metabolites in more than half of the samples within the group, and filling null values with K-Nearest Neighbor method. After the original data were converted to mzXML format by ProteoWizard, the XCMS package for R (v4.1.2) was used for peak identification, filtration, and alignment to acquire the mass-to-charge ratio, retention time, and peak area based on positive and negative ion modes. The parameters in XCMS were set as follows: centwave settings for feature detection (∆m/z = 25 ppm, peakwidth = c (10, 60)), obi-warp settings for retention time correction (profStep = 1), and parameters for chromatogram alignment (minfrac = 0.5, bw = 5, and mzwid = 0.025). The metabolites identification was based on accurate mass matching (<25 ppm) and secondary spectrum matching methods and search of the laboratory self-built commercial database by using the annotation level described by the metabolic standards initiative (Members et al., 2007). The MS/MS spectra similarity score was calculated using the forward dot-product algorithm (Stein and Scott, 1994), which considers both fragments and intensities, and the score threshold is 0.6. Thermo mzVault (version 2.3) was utilized to create a mass spectral library and Tracefinder (version 4.1) was adopted for quantification.

### 2.8 Statistical analysis

The log transformation (base 10) was applied to normalize the data. Data scaling was performed using mean-centered and was divided by standard deviation of each variable. After the obtained data were normalized, principal component analysis (PCA) and orthogonal projection to latent structures-discriminant analysis (OPLS-DA) were performed to determine differences in metabolites between the two groups using MetaboAnalyst 5.0 (https://www.metaboanalyst.ca/). Differential metabolites were screened by variable importance in projection (VIP) scores of the OPLS-DA model and statistical significance according to the Student’s t-tests.

Spearman rank correlation analysis was used to determine the correlation between differential metabolites and bone quality parameters. Differential metabolites with correlation coefficients higher than 0.5 and *p*-values less than 0.05 were imported into MetaboAnalyst 5.0 to perform pathway analysis to identify metabolic pathways associated with bone quality.

All bone quality data were analyzed using SPSS statistical software (version 25.0). Results were expressed as mean ± standard deviation (SD). *p* < 0.05 was considered to be a statistically significant difference.

## 3 Results

### 3.1 Body weight and tibia morphology parameters from the two rearing systems

**FIGURE 1:**
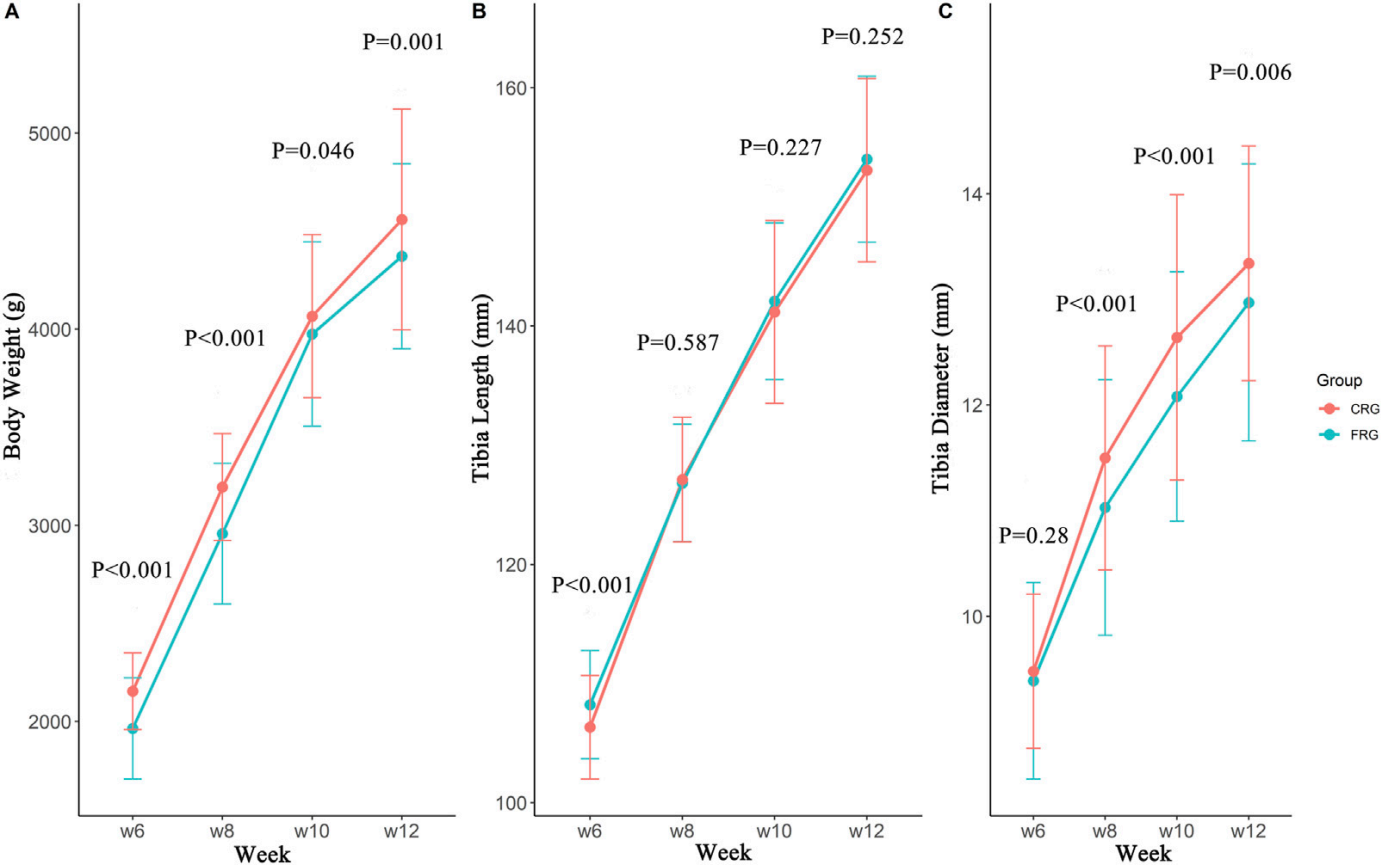
The developmental differences of the tibia at different periods between two groups. The body weight **(A)**, tibia length **(B)**, and tibia diameter **(C)** development of cage-reared and floor-reared chickens from 6 to 12 weeks of age. *n* (CRG) = 246, *n* (FRG) = 173.

As shown in Figure 1A, the body weight of the CRG was significantly higher than that of the FRG at 6, 8, 10, and 12 weeks of age (*p* < 0.05). Compared with CRG, the tibia length of FRG at 6 weeks was greater (*p* < 0.01); however, there were no significant differences at 8, 10, or 12 weeks between the two flocks (Figure 1B). The results also indicated that the CRG group exhibited a wider tibia diameter than FRG at 8, 10, and 12 weeks (*p* < 0.01, Figure 1C).

### 3.2 Bone quality parameters from the two rearing systems

The results for related bone quality parameters indicated that cage rearing systems significantly decreased BMD, ash content, calcium content, and phosphorus content of the tibia and femur (*p* < 0.05, Table 1). The stiffness and Young’s modulus of the femur in the FRG were significantly higher than in the CRG (*p* < 0.05). However, there were no significant differences in BMC or BBS between the two groups.

**TABLE 1:** Effect of different rearing systems on bone quality parameters.

Traits	Tibia			Femur		
	CRG	FRG	*p*-value	CRG	FRG	*p*-value
BMD, g/cm^2^	0.28 ± 0.06	0.33 ± 0.02	0.030	0.24 ± 0.07	0.29 ± 0.03	0.021
BMC, g	6.81 ± 1.75	7.94 ± 0.70	0.050	3.89 ± 1.39	4.79 ± 0.59	0.052
BBS, N	228.47 ± 163.10	190.92 ± 126.06	0.536	352.95 ± 132.05	342.01 ± 49.62	0.791
Stiffness, kN	120.83 ± 32.65	114.70 ± 22.63	0.598	205.78 ± 48.87	253.35 ± 59.69	0.044
YM, GPa	3.74 ± 1.01	3.55 ± 0.70	0.598	3.00 ± 0.71	3.69 ± 0.87	0.044
Ash content, %	43.15 ± 1.35	46.33 ± 1.53	<0.001	45.05 ± 1.7	48.61 ± 2.22	<0.001
Calcium content, %	15.36 ± 1.00	17.17 ± 0.91	<0.001	13.73 ± 0.73	16.12 ± 1.09	<0.001
Phosphorus content, %	7.15 ± 0.45	8.27 ± 0.39	<0.001	6.15 ± 0.48	6.93 ± 0.54	0.001

All values are reported as mean ± SD, *n* = 12. BMD, bone mineral density; BMC, bone mineral content; BBS, bone breaking strength; YM, Young’s modulus; CRG, cage rearing group; FRG, floor rearing group.

### 3.3 Quality control and multivariate statistical analysis of the metabolomics assay

**FIGURE 2:**
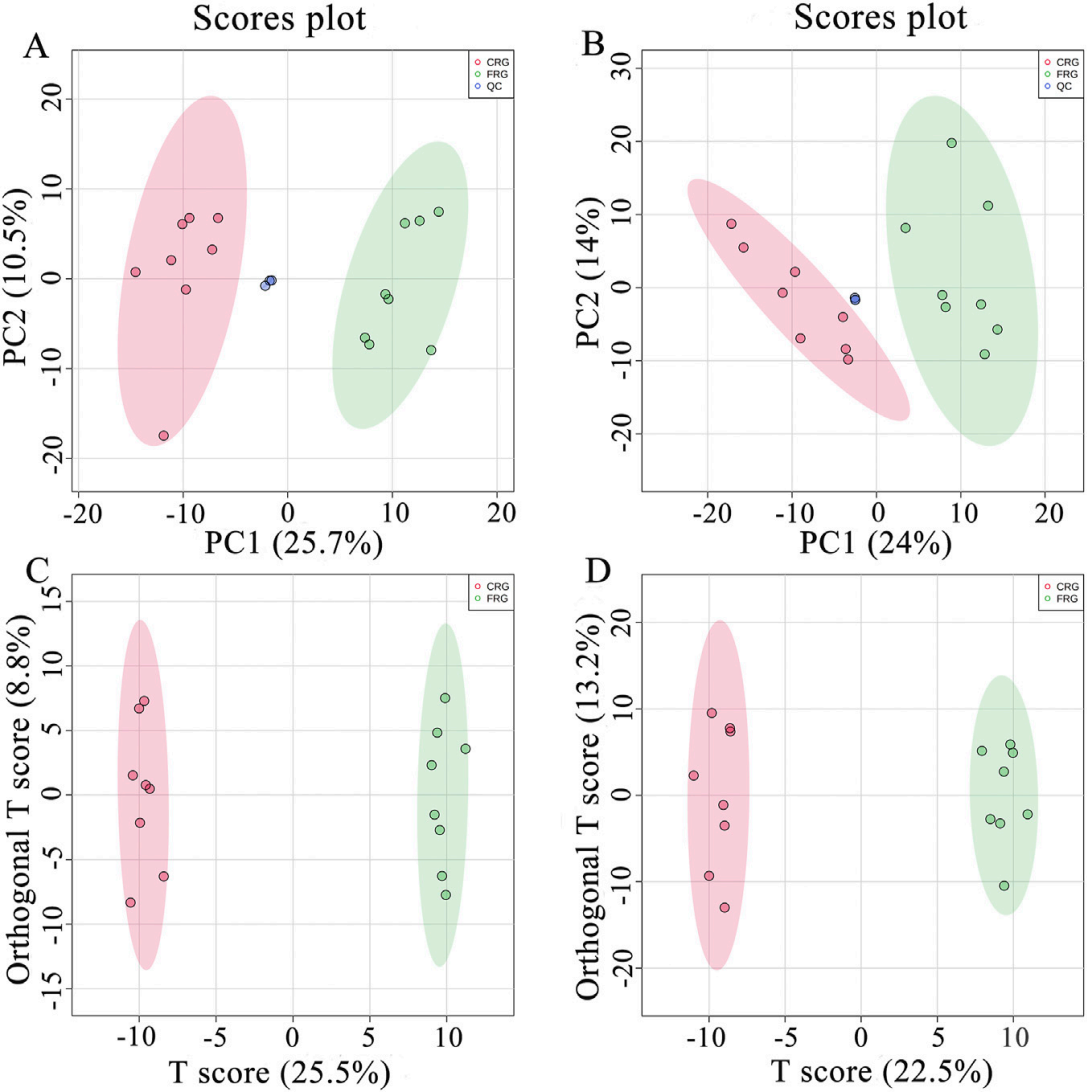
Multivariate statistical analysis of the metabolomics assay in positive and negative ion modes. **(A)** PCA score plot for the metabolomics assay in positive ion mode. **(B)** PCA score plot for the metabolomics assay in negative ion mode. **(C)** OPLS-DA score plot for the metabolomics assay in positive ion mode. **(D)** OPLS-DA score plot for the metabolomics assay in negative ion mode.

As shown in Figures 2A,B, the close clustering of QC samples demonstrated that this method was stable and repeatable. Moreover, the clustering analysis showed that the metabolic profile of the CRG group was clearly separated from that of the FRG group in both positive and negative ion modes, indicating that the housing systems affected serum metabolites. All serum metabolic profiles were utilized to construct a supervised OPLS-DA model to identify differential metabolites, and OPLS-DA score plots indicated that the sample clustering of CRG was clearly divergent from FRG (Figures 2C,D). The model parameters are shown in Supplementary Figure S1.

### 3.4 Screening and correlation analysis of differential metabolites

**FIGURE 3:**
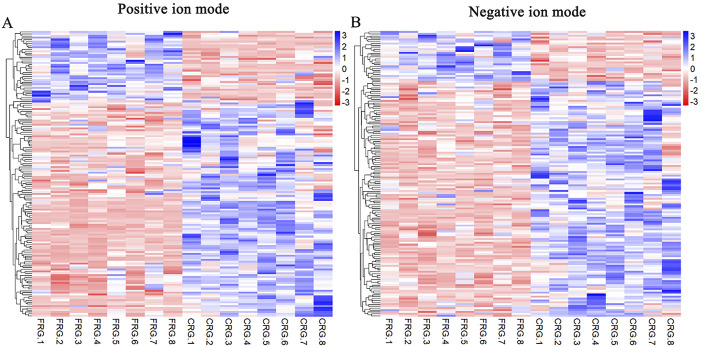
Analysis of annotated differential metabolites from two housing systems. Hierarchical clustering heatmap of differential metabolites in positive **(A)** and negative **(B)** ion modes. Blue and red indicate high and low concentrations in cage-reared birds, respectively. Each row represents one metabolite.

The analysis detected 399 and 422 compounds in positive and negative ion modes, respectively. We integrated the VIP values of the OPLS-DA model and *p*-values of the *t*-test to screen for differential metabolites between the two groups, and the threshold criteria were set to VIP ≥1 and *p* < 0.05. The total number of annotated differential metabolites between the FRG and CRG were 148 and 149 in positive and negative ion modes, respectively, and the results are shown in Supplementary Table S2. There were 112 compounds had higher concentrations, and 36 had lower concentrations from CRG compared with FRG in positive ion mode (Figure 3A). Meanwhile, 123 compounds had greater levels, and 26 had lower levels from CRG compared with FRG in negative ion mode (Figure 3B).

### 3.5 Identification of differential metabolites correlated with bone quality parameters

Spearman rank correlation analysis was employed to calculate correlation coefficients between differential metabolites with bone quality parameters of the tibia and femur. The correlation coefficients and hypothesis tests for each correlation analysis are shown in Supplementary Table S3. We found 257 differential metabolites correlated with 16 bone quality parameters. Among these, 60 metabolites were strongly correlated with bone quality parameters (| correlation coefficient | > 0.80, and *p* < 0.05), including leucine, myristoleic acid, glycocholic acid, N-phenylacetamide, and hypoxanthine; 197 metabolites were weakly correlated with bone quality parameters (| 0.50 < correlation coefficient | < 0.80, and *p* < 0.05).

### 3.6 Metabolic pathway perturbation affecting bone quality parameters

To further elucidate the potential metabolic pathways affecting bone quality parameters, the above differential metabolites associated with bone quality parameters were imported into MetaboAnalyst 5.0 for pathway analysis. A total of 45 metabolic pathways were obtained, and a summary of the results is provided in Supplementary Table S4. The metabolic pathways with an impact value exceeding 0.1 were considered as significantly relevant pathways according to previous research (Liu et al., 2018; Tan et al., 2018). The pathway analysis indicated that 15 metabolic pathways were responsible for bone quality parameters, with six amino acid metabolism pathways (histidine metabolism, phenylalanine metabolism, phenylalanine, tyrosine and tryptophan biosynthesis, alanine, aspartate and glutamate metabolism, cysteine and methionine metabolism, and tryptophan metabolism), two lipid metabolism pathways (glycerophospholipid metabolism and primary bile acid biosynthesis), two carbohydrate metabolism pathways (TCA cycle and ascorbate and aldarate metabolism), one nucleotide metabolism pathway (pyrimidine metabolism), and others (D-glutamine and D-glutamate metabolism, terpenoid backbone biosynthesis, porphyrin and chlorophyll metabolism, and taurine and hypotaurine metabolism) (Table 2).

**TABLE 2:** The pathway analysis for annotated differential metabolites.

Pathway name^a^	Hits^b^	Impact value^c^	Raw p
Phenylalanine, tyrosine and tryptophan biosynthesis	1	0.5000	0.280
Phenylalanine metabolism	2	0.5000	0.126
d-Glutamine and D-glutamate metabolism	1	0.5000	0.389
Taurine and hypotaurine metabolism	2	0.4286	0.126
Alanine, aspartate and glutamate metabolism	3	0.4231	0.381
Glycerophospholipid metabolism	7	0.4119	0.017
Tryptophan metabolism	5	0.3369	0.188
Ascorbate and aldarate metabolism	1	0.2500	0.561
Pyrimidine metabolism	6	0.2167	0.089
Histidine metabolism	3	0.1885	0.126
Porphyrin and chlorophyll metabolism	3	0.1649	0.425
Citrate cycle (TCA cycle)	3	0.1240	0.205
Terpenoid backbone biosynthesis	1	0.1143	0.774
Primary bile acid biosynthesis	3	0.1140	0.718
Cysteine and methionine metabolism	1	0.1045	0.935

^a^ Pathway name, name of KEGG, pathways.

^b^ Hits, number of KEGG, pathways containing differentiated metabolites.

^c^ Impact value, the pathway impact value calculated from pathway topology analysis.

## 4 Discussion

The present work aimed to study the effects of different rearing systems on bone quality parameters in meat-type chickens. Here, cage-reared chickens displayed higher body weight than floor-reared chickens during the same growth period (*p* < 0.05). Similar to our results, broilers housed in a multilayer cage rearing system had greater body weight gain than chickens raised in a floor litter rearing system from 29 to 42 days of age (Li et al., 2017). Birds grown in floor pens had greater tibia lengths than cage-reared birds suggested by previous studies (Bond et al., 1991). Differences in tibial morphology may be due to mechanical pressures caused by body weight. We found that cage-reared chickens have wider tibiae and greater body weight.

Previous studies have compared skeletal biological properties of chickens reared under different systems and demonstrated that the chickens raised in the conventional cages had poor bone quality (BMD, BMC, BBS, and bone ash) compared with chickens housed in floor, free-range, or aviary systems (Newman and Leeson, 1998; Regmi et al., 2016; Yue et al., 2020; Sharma et al., 2021). Our results also indicated that the BMD, ash content, calcium content, and phosphorus content of the tibia and femur of the FRG were significantly higher than those of the CRG broilers, and these traits were positively correlated (Supplementary Figure S2). Although the differences in BMC of the tibia and femur were not significant between the two groups, a similar trend between BMC and BMD suggested that the rearing system altered bone status. This might be due to the fact that chickens raised in CRG had greater space allowance to walk, run, jump, and wing-flap. In this experiment, the femur of floor reared-birds had significantly higher Young’s modulus (*p* < 0.05), indicating that the bone was less susceptible to deformation. Surprisingly, the decreased bone breaking strength in the FRG was observed in our study, a result that was contradictory with previous research (Leyendecker et al., 2005; Yilmaz Dikmen et al., 2016). We speculate that this may be not caused by total bone mass, but by its geometrical distribution, porosity, and structural organization.

To illustrate the effect of different housing systems on bone quality, serum metabolite profiling was performed to identify differential metabolites in the chickens. In this study, a total of 297 differential metabolites were detected, among which 60 were strongly associated with 16 bone quality parameters, and 197 metabolites were weakly correlated with 16 bone quality parameters. These compounds have been shown to be related to bone quality in numerous studies. Histamine is one of the potential endogenous factors affecting bone remodeling, as it acts as a mediator of allergic reactions and as a neuromodulator that induces the production of gastric acid. Excessive release of histamine in mastocytosis and allergic diseases can lead to the development of osteoporosis (Wiercigroch and Folwarczna, 2013). Previous studies have found that the histamine and histamine H4 receptors promote osteoclastogenesis in rheumatoid arthritis (Kim et al., 2017). N-acetylmannosamine was significantly more abundant in the postmenopausal osteoporosis group and was negatively correlated with BMD (He et al., 2020). Inosine was significantly associated with low BMD status in the discovery set and successfully validated in the independent replication set (Mei et al., 2020). In our study, the concentrations of histamine, N-acetylmannosamine, and inosine were higher in cage-reared chickens, a result that may be associated with poor bone quality. Berberine can exert a potent bone protective effect by promoting bone formation and inhibiting bone resorption and fat accumulation in bone marrow (Chen et al., 2021). Recent studies have suggested that stachydrine can be a potential option for treating osteoclast-related diseases due to its inhibitory effect on osteoclastogenesis both *in vivo* and *in vitro* (Meng et al., 2019). Our experiment found that the levels of berberine and stachydrine increased in the serum of floor-reared chickens, suggesting that these compounds may be associated with normal bone development.

Continuous bone remodeling *via* formation and resorption is an essential process for maintaining bone homeostasis and is closely associated with energy metabolism. Energy in the form of adenosine triphosphate (ATP) is generated from proteins, fats, and carbohydrates through a range of metabolic pathways (Suzuki and Iwata, 2021). In addition, an accumulating number of studies suggest that amino acids are beneficial for collagen formation and osteoblast differentiation (Chevalley et al., 1998; Ding et al., 2018). A total of six pathways of amino acid metabolism were found to be connected with bone quality parameters in this study. Phenylketonuria is a disorder caused by abnormal phenylalanine metabolism in which a particularly high concentration of phenylalanine causes lower bone mineral density in patients compared with control subjects (Barta et al., 2017). There is evidence that histidine metabolism is one of the most relevant metabolic pathways related to the dysregulation of 94 metabolites in patients with low BMD (Aleidi et al., 2021). In another study, several serum metabolite pathways were associated with both lumbar spine and femoral neck osteoporosis, including phenylalanine, tyrosine and tryptophan biosynthesis, alanine, aspartate and glutamate metabolism, and cysteine and methionine metabolism. In addition, tryptophan metabolism was correlated with both lumbar spine and femoral neck osteoporosis in a study using fecal metabolites profiling (Ling et al., 2021). Both of the above studies and the present study demonstrated that amino acid metabolism is critical for bone development.

In addition to storing energy, lipids are an essential component of bone (mainly present in bone marrow). The mesenchymal stem cells in bone marrow can differentiate into osteoblasts, adipocytes, chondrocytes, and other cell types, indicating that there is a definite relationship between lipid metabolism and bone quality. Several lines of evidence have suggested that excessive lipid accumulation is associated with the deterioration of bone. Total cholesterol, low-density lipoprotein cholesterol, and high-density lipoprotein cholesterol were negatively associated with lumbar spine BMD in postmenopausal women (Zhang et al., 2020). Obesity induced by a high-fat diet decreased cancellous bone mass in mice (Cao et al., 2009). The latter study also reported that a high-fat diet induced bone loss in juvenile mice, primarily due to elevated osteoclast bone resorption and improved expression of osteoclastogenic regulators including RANKL, THF, and PPAR-gamma by affecting the bone marrow microenvironment (Shu et al., 2015). This may explain the negative correlation between compounds enriched in lipid metabolism and bone quality (Supplementary Table S3). Researchers have found that metabolites associated with whole-body BMD Z-scores were partially enriched in fatty acid related-metabolic pathways, including glycerophospholipid metabolism, glycosphingolipid metabolism, and fatty acid activation and biosynthesis, similar to our findings (Bellissimo et al., 2021). In our study, the concentrations of compounds enriched in lipid metabolism were significantly higher in the CRG group, suggesting that these metabolites have a certain effect on bone metabolism.

The remaining metabolic pathways may also play important roles in bone development. Bellissimo et al. (2020) employed ultra-high-resolution metabolomics (HRM) to determine plasma metabolic pathways related to bone turnover markers, and they found that metabolites significantly associated with procollagen type Ⅰ N-terminal propeptide (P1NP) and C-terminal telopeptides of type Ⅰ collagen (CTX) were enriched in pathways linked to the TCA cycle, amino acid metabolism, and lipid metabolism, indicating that lipids, amino acid, and carbohydrates were related to bone turnover markers. The effect of bone marrow mesenchymal stem cell transplantation on metabolic pathways within osteoporotic bone was associated with taurine and hypotaurine metabolism, arachidonic acid metabolism, and pentose and glucuronate interconversion (Wang et al., 2022). Postmenopausal women with low BMD were characterized by elevated glutamine levels compared to those with high BMD, indicating that D-glutamine and D-glutamate metabolism is a potential metabolic pathway that affects BMD (You et al., 2014). The above studies and the results of this study suggest that these pathways play a certain role in bone development, but the specific mechanisms need to be further studied.

## 5 Conclusion

In summary, cage-reared chickens had wider tibiae and greater body weight than floor-reared chickens. The bone mineral density, ash content, calcium content, and phosphorus content of the tibia and femur, stiffness and Young’s modulus of the femur of floor-reared chickens were significantly higher than those of CRG, but there were no significant differences in bone breaking strength or mineral content of the tibia and femur. Taken together, these results suggest that the cage rearing system has a deteriorative effect on bone quality parameters. We identified 257 differential metabolites and 15 metabolic pathways were responsible for bone quality parameters based on untargeted metabolomics and pathway analysis.

## Data Availability

The raw data supporting the conclusion of this article will be made available by the authors, without undue reservation.
