# Supplementary figures and images for: Untargeted metabolomics reveals the effect of rearing systems on bone quality parameters in chickens
Source: Front Genet. 2023 Jan 4;13:1071562. doi: 10.3389/fgene.2022.1071562 (PMC9846032; doi:10.3389/fgene.2022.1071562)

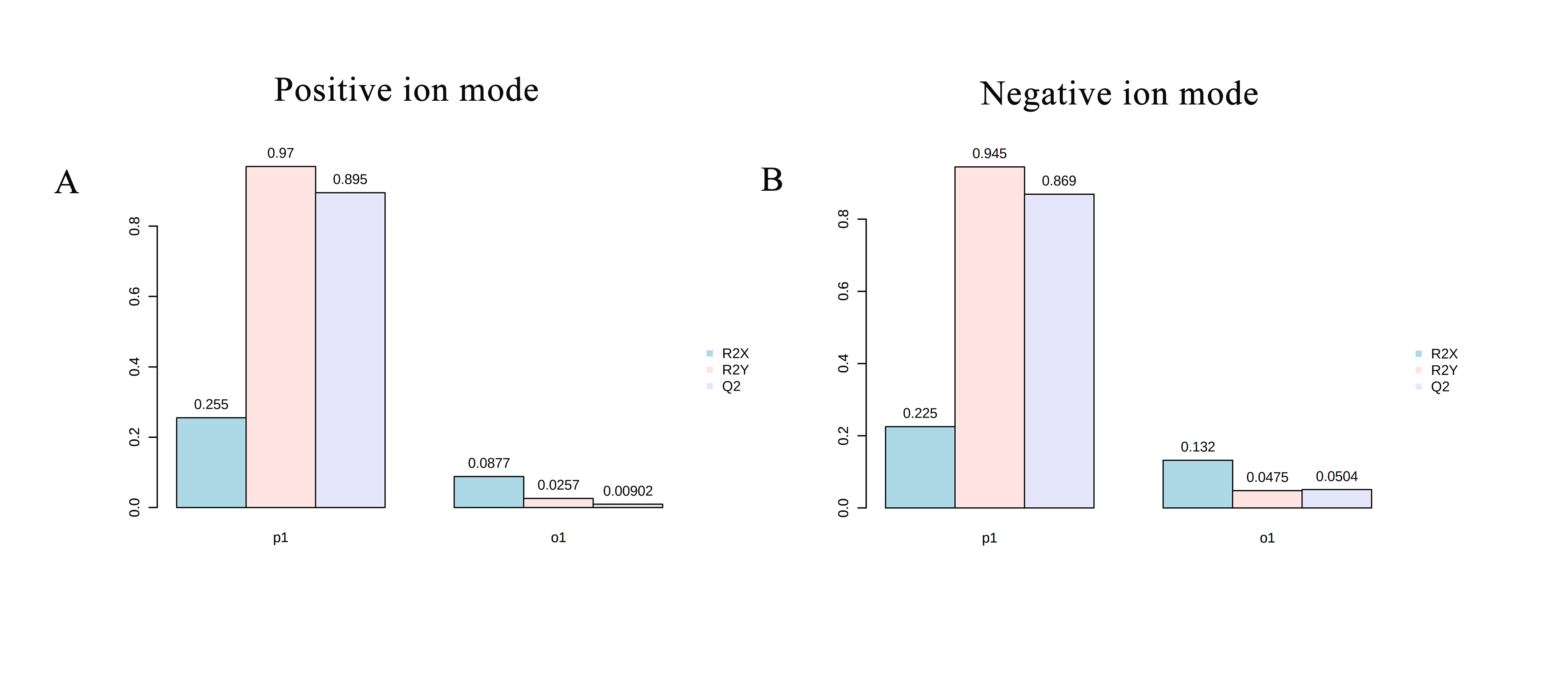

Supplement: Supplementary file 3 [file Image1.JPEG]

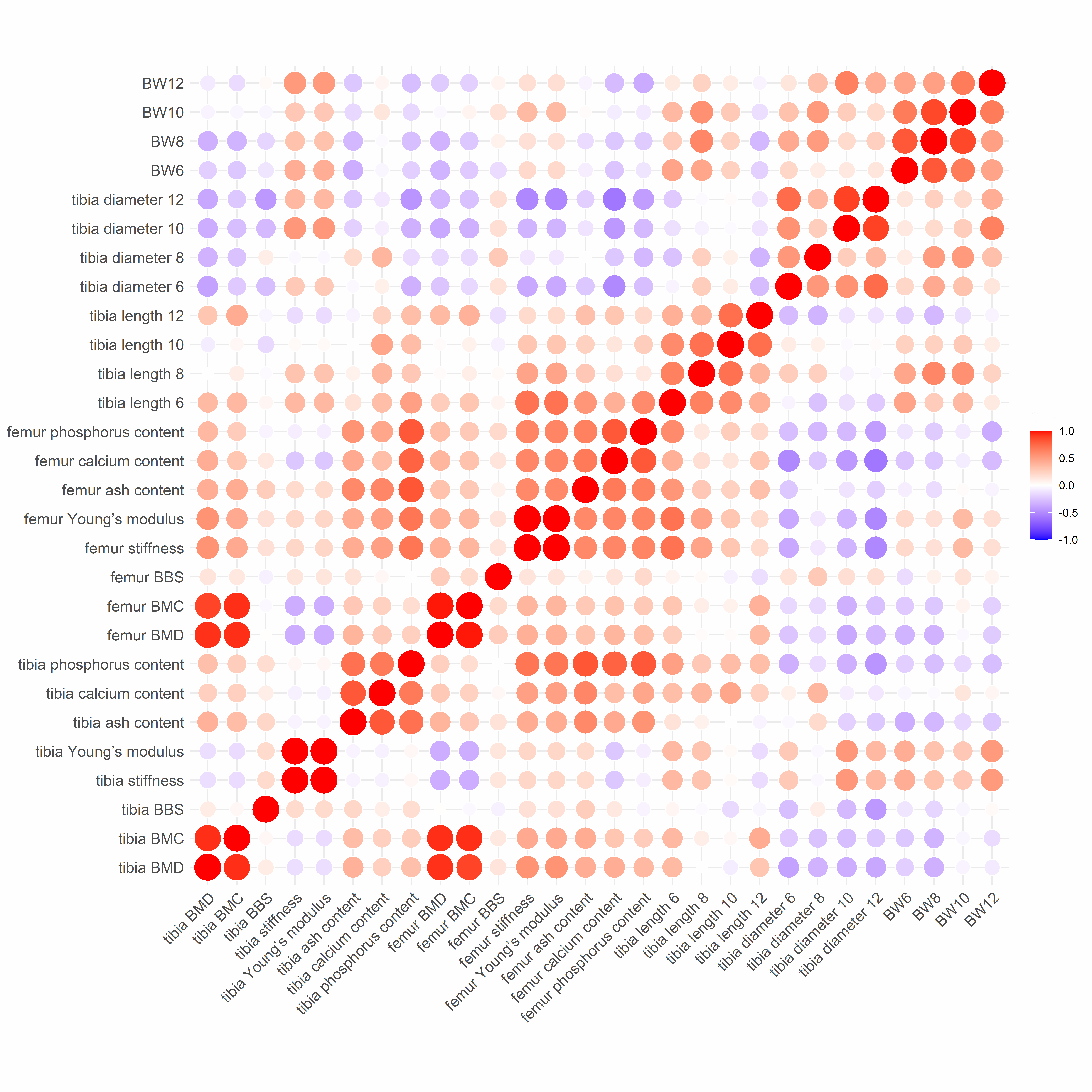

Supplement: Supplementary file 4 [file Image2.JPEG]
